# PKCα promotes the mesenchymal to amoeboid transition and increases cancer cell invasiveness

**DOI:** 10.1186/s12885-015-1347-1

**Published:** 2015-04-29

**Authors:** Katarína Vaškovičová, Emilia Szabadosová, Vladimír Čermák, Aneta Gandalovičová, Lenka Kasalová, Daniel Rösel, Jan Brábek

**Affiliations:** 1 Department of Cell Biology, Laboratory of Cancer Cell Invasion, Charles University in Prague, Prague, Czech Republic; 2 Current affiliation: Microscopy Unit, Institute of Experimental Medicine, The Czech Academy of Sciences, Prague, Czech Republic

**Keywords:** Amoeboid, Mesenchymal, Plasticity, PKCα, Invasiveness, Metastasis

## Abstract

**Background:**

The local invasion of tumor cells into the surrounding tissue is the first and most critical step of the metastatic cascade. Cells can invade either collectively, or individually. Individual cancer cell invasion can occur in the mesenchymal or amoeboid mode, which are mutually interchangeable. This plasticity of individual cancer cell invasiveness may represent an escape mechanism for invading cancer cells from anti-metastatic treatment.

**Methods:**

To identify new signaling proteins involved in the plasticity of cancer cell invasiveness, we performed proteomic analysis of the amoeboid to mesenchymal transition with A375m2 melanoma cells in a 3D Matrigel matrix.

**Results:**

In this screen we identified PKCα as an important protein for the maintenance of amoeboid morphology. We found that the activation of PKCα resulted in the mesenchymal-amoeboid transition of mesenchymal K2 and MDA-MB-231 cell lines. Consistently, PKCα inhibition led to the amoeboid-mesenchymal transition of amoeboid A375m2 cells. Next, we showed that PKCα inhibition resulted in a considerable decrease in the invading abilities of all analyzed cancer cell lines.

**Conclusions:**

Our results suggest that PKCα is an important protein for maintenance of the amoeboid morphology of cancer cells, and that downregulation of PKCα results in the amoeboid to mesenchymal transition. Our data also suggest that PKCα is important for both mesenchymal and amoeboid invasiveness, making it an attractive target for anti-metastatic therapies.

**Electronic supplementary material:**

The online version of this article (doi:10.1186/s12885-015-1347-1) contains supplementary material, which is available to authorized users.

## Background

The ability to form metastases is the most dangerous property that tumor cells can acquire. Cells of a primary tumor can disseminate throughout the body and potentially establish secondary tumors – metastases - in a process called the metastatic cascade (reviewed in [1]). The local invasion of tumor cells into the surrounding tissue is the first and most critical step of the metastatic cascade, and importantly, it determines the metastatic potential of many tumor cell types. Cells can invade through tissue and the extracellular matrix (ECM) either collectively, or individually. During collective invasion, the cell – cell adhesions between cells remain intact and cells migrate as a group in the form of strands, tubes, sheets or irregular masses [2-4]. Individual invasion is the invasion of single cells and can occur in mesenchymal or amoeboid mode (reviewed in [5,6]). The mesenchymal mode of invasion can be recognized by the typical fibroblast-like morphology of individually-invading cancer cells and also by their polarized character. At the leading edge, the cells generate actin rich structures, filopodia and lamellipodia, that trigger the cancer cell movement. Formation of filopodia and lamellipodia is regulated by the small GTPases Rac1 and Cdc42 [7,8]. Mesenchymal invasion is also dependent on local degradation of the ECM by degrading enzymes. The secretion of proteolytic enzymes is localized in actin-rich adhesion structures called invadopodia [9].

The morphology of amoeboid cells is typically round or ellipsoid in a 3D environment. Amoeboid cancer cell invasion is mediated by the contractions of cortical actin, which is regulated by the Rho/ROCK signaling pathway. Two types of Rho GTPase molecules, RhoA and RhoC, activate ROCK kinase. ROCK kinase phosphorylates MLCP (myosin light chain phosphatase) to inhibit its phosphatase function towards the myosin light chain (MLC), and ROCK therefore increases MLC [10-12]. To promote the effect, MLC2 is also directly phosphorylated by ROCK kinase. The phosphorylation of MLC leads to the generation of higher contractile forces by the actomyosin cortex, thus allowing the migration of cancer cells through ECM fibers independently of proteolytic degradation [13,14].

Cancer cell invasion is a very complex and plastic process, and the mesenchymal and amoeboid modes of invasion are mutually interchangeable. Activation or inhibition of specific signaling cascades leading to a specific mode of invasion can cause a switch from one invasion mode to another (reviewed in [5,6,15,16]). It has been demonstrated that the mesenchymal-amoeboid transition (MAT) may be an escape mechanism in tumor cell invasion after the abolition of pericellular proteolysis [17]. The mechanisms of MAT or the amoeboid-mesenchymal transition (AMT) are, however, poorly understood. Only a limited number of studies describing the molecular mechanisms underlying MAT/AMT have been published so far (reviewed in [6]). In order to better understand the plasticity of individual cancer cell invasion, it is critical to identify other proteins involved in MAT and/or AMT.

To identify new signaling proteins involved in MAT/AMT, we performed proteomic analysis of AMT with melanoma cells cultured in a 3D Matrigel matrix. Protein microarrays were chosen instead of gene expression microarrays because AMT and MAT are highly dynamic processes and thus are mostly defined by changes in posttranslational modifications of proteins and not in mRNA expression levels. To our knowledge, this is the first proteomic study of this kind performed with cells in a 3D matrix. We identified PKCα as a protein important in the amoeboid mode of cancer cell invasion. Next, using biochemical and genetic approaches, we confirmed the role of PKCα in regulating cancer cell morphology, the effectiveness of cancer cell invasiveness, and overall cancer cell plasticity.

## Methods

### Cells, culture and material

A375m2 melanoma cells were cultivated in full DMEM medium: DMEM (GIBCO) with 4500 mg/l L-glucose, L-glutamine, and pyruvate, supplemented with 10% fetal bovine serum (Sigma), 2% antibiotic-antimycotic (GIBCO) and 1% MEM non-essential amino acids (GIBCO). MDA-MB-231 breast cancer cells were maintained in full RPMI medium: RPMI (Gibco) supplemented with 10% fetal bovine serum (Sigma), 2% antibiotic-antimycotic (GIBCO) and 1% MEM non-essential amino acids (GIBCO). K2 sarcoma cells were cultivated in in MEM with Hanks’ salts supplemented with 10% bovine serum (ZVOS), 0.09% sodium bicarbonate, 0.12 g/L sodium pyruvate, and 1 mmol/L glutamine. All cell lines were kept at 37°C in a humidified atmosphere with 5% CO_2_. Gö6976 – PKCα and PKCβ inhibitor, and PMA (phorbol 12-myristate 13-acetate) – PKC activator were used at concentrations of 1 μM and 162nM, respectively.

### DNA and RNA constructs and transfection

Flag-tagged wt PKCα, constitutive active form (with myristoylation sequence from Src) and dominant-negative PKCα (K368R) in pCMV6 vector were all obtained from Addgene Inc. Silencer® Select Validated siRNA against PKCα was obtained from Applied Biosystems and a final concentration of 10 nM was used for transfections. All transfections were performed using jetPRIME™ transfection kits (Polyplus Transfection) according to the manufacturer’s instructions.

### Immunoblotting

For 2D lysates, cells were cultured on plates with or without Y 27632 for 48 hours. then washed with phosphate buffered saline (PBS) and lysed in modified RIPA buffer (0.15 M NaCl; 50 mM Tris HCl (pH 7.4); 1% Nonidet P 40; 0.1% SDS; 1% sodium deoxycholate; 5 mM EDTA; 50 mM NaF ).

For 3D lysates, cells were first embedded into 500 μl of Matrigel solution (BD, MatrigelTM Basement Membrane Matrix Phenol Red Free) with or without Y 27632. After 24 hours, Matrigel was solubilized using Cell Recovery Solution (BD) for 30 min at 4°C. Cells were washed with PBS and lysed in RIPA buffer.

Protein concentrations in the lysates were determined using the DC Protein Assay (Bio Rad). Protein lysates were diluted in Laemmli sample buffer (0.35 M Tris HCl, pH 6.8; 10% SDS; 40% glycerol; 0.012% bromphenol blue). For immunoblotting, samples were separated on 10% SDS polyacrylamide gels and transferred onto nitrocellulose membranes. Nonspecific activity was blocked by incubating membranes for 1 h at 37°C in Tris buffered saline containing 4% bovine serum albumin. Membranes were then incubated overnight at 4°C with primary antibody, washed extensively with TTBS, and then incubated for 1 h at room temperature with HRP conjugated secondary antibody. After extensive washing in TTBS, the blots were developed using the LAS 1000 Single System (Leica). Monoclonal antibodies against FLAG anti-FLAG (F3165) and total PKCα (P 4334) were from Sigma Aldrich Inc., PKCα pSer657 (sc-12356) and actin (sc 1616) were from Santa Cruz Biotechnology Inc., β-catenin antibody (610154) was from BD Transduction laboratories, p-cofilin Ser3 antibody (clone 77G2) was from Cell Signaling Technology. Secondary Antibodies GAR-HRP polyclonal (sc 2030) and DAG-HRP polyclonal (sc 2033) were from Santa Cruz Biotechnology Inc., and GAM-HRP polyclonal (35502) was from Thermo Fisher Scientific Inc.

### Protein microarrays

2.4×10^6^ cells were embedded into 8 ml of Matrigel (BD, MatrigelTM Basement Membrane Matrix Phenol Red Free) in the presence or absence of 50 μM Y 27632. After 24 hours of incubation the Matrigel was solubilized with Cell Recovery Solution (BD) for 30 min at 4°C. Cells were sedimented by centrifugation, washed in PBS and sent for analysis by Kinexus Bioinformatics Corporation (EXPANDED KINEX™ 800 ANTIBODY MICROARRAY SERVICE) on dry ice to determine changes in the expression and/or phosphorylation status of selected signaling proteins. Two experiments were performed in duplicates.

### Cell morphology assays in 3D collagen

To analyze cell morphology of whole cell population in 3D collagen (in invasion assays only 10% of cells reach deeper layers of 3D collagen), cells were trypsinized, washed in complete medium, counted, and then 10^5^ cells were mixed with 500 μl of 3 mg/ml Collagen R (Serva) in complete medium. This suspension of cells in collagen (500 μl) was loaded into a well of a 24-well plate, the gel was allowed to polymerize at 37°C for 30 min, and was then overlaid with complete medium. After 24 hours, the morphology of cells in 3D collagen was analyzed using a Nikon Eclipse TE2000-S microscope (20×/0,40 HMC objective). Morphology of each cell was determined to be either elongated, intermediate or round. This was done on the basis of an elongation index. Elongation index was calculated as the length of the cell divided by the width. Cells whose elongation index was greater than 2 were considered elongated. Intermediate cells had an elongation index of 1,5-2; for rounded cells, the index was 1-1,5. Dividing cells were excluded from the analysis. Proportion of elongated, intermediate and rounded cells was calculated and expressed percentually. Three independent experiments (at least 300 cells per experiment) were analyzed for each condition. As the data have the form of counts in categories, the Pearson’s Chi-squared test was used to reveal statistically significant differences.

### In vitro cell invasion assays in 3D collagen

The 3D collagen invasion assay was analyzed as described previously [18]. Briefly, cell suspension (1 × 10^5^ cells/ml) was added on top of a collagen gel in a multiwell plate (μ-Slide Angiogenesis ibiTreat Microscopy Chamber (IBIDI)), and after 48 hours the level of invasion was measured. Pictures of 3D collagen cross-sections in the selected view were taken every 10 μm using a Nikon-Eclipse TE2000-S (20×/0.40 HMC objective) and NIS-Elements software. For each experiment, invasion was analyzed in 3 wells and average invasion depth was assessed in 6 fields of view per individual well. In order to compare individual experiments, the average invasion depth was normalized to that of untreated cells. In average the depth of at least 10 um was achieved by 43 percent of K2 cells, 50 percent of MDA-MB-231 cells and 60 percent of A375m2 cells. Three independent experiments were analyzed for each condition. The significance of differences was analyzed with ANOVA followed by Tukey’s honest significant difference test. The analysis was performed in R [19].

### Gelatin degradation assay

Dry coverslips were coated with a thin layer of FITC-conjugated gelatin and immediately overturned on a drop of 0.5% ice-cold glutaraldehyde in PBS for 15 min incubation in the dark. Coverslips were then transferred to a 12-well plate, with the coated side up and gently washed three times with PBS, incubated with sodium borohydride (5 mg/ml) in PBS for 3 min and then finally washed with PBS. The cover slips were sterilized in 70% ethanol for 1 min, dried for 10 min in a sterile hood and quenched in complete medium for 1 h at 37°C. 48 hours after transfection, cells were plated onto prepared FITC-labeled gelatin-coated coverslips. Overnight, cells were allowed to adhere and degrade gelatin and next day, cells were fixed and stained for actin. For actin staining, fluorescently labeled phalloidin (Alexa Fluor® 405 phalloidin (Invitrogen)) was used. Finally, coverslips were mounted on a mounting slide. Images for gelatin degradation assay were taken by fluorescent microscope Nikon Eclipse TE2000-S using (20×/0.40 HMC objective). Seven random fields were analyzed in each experiment using AnalyzeGelStack software (obtained from Prof. R. Buccione). Using this software, degradation area normalized to the number and area of cells (degradation area per cell) was measured. Values were normalized to value of control untreated cells. Confocal microscope Leica DM IRE2 was used for better cell visualization and taking representative pictures.

### Cell viability assay

Cell viability was assessed by the trypan blue dye exclusion method. Briefly, cells were seeded into two 3.5 mm dishes, the next day 1 μM Gö6976 was added to one of the dishes. After 48 hours cells were trypsinized and diluted 1:1 in 0.4% sterile filtered trypan blue solution. The cells were incubated in the trypan blue solution for 5 minutes at room temperature and then transferred onto a haemocytometer and counted under a light microscope. Viable cells appeared bright, dead cells were blue. Three independent experiments were performed, each time both sides of the haemocytometer were counted twice for both conditions. To assess the statistical significance of the differences, the Pearson’s Chi-squared test was performed.

## Results

### Protein microarray analysis reveals the down-regulation of PKCα phosphorylation in highly metastatic amoeboid A375m2 melanoma cells after AMT

**Figure 1:**
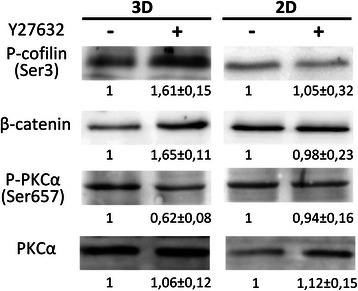
Proteins with differential expression/phosphorylation after AMT in A375m2 cells (Kinexus). Phosphorylation of cofilin2 at Ser3, expression of β-catenin, phosphorylation of PKCα at Ser657 and expression of PKCα were detected using corresponding antibodies in cell lysates from A375m2 strain upon AMT (induced by ROCK kinase inhibitor Y37632) both in 3D and 2D environment. Representative immunoblots are shown. Densitometric analysis was made using ImageJ software. Quantification was made from three independent experiments and normalized to that of untreated cells.

To identify new proteins involved in amoeboid invasiveness and the AMT, we analyzed proteomic profiles of cells before and after AMT. We used amoeboid melanoma A375m2 cells that were cultivated in 3D Matrigel and either induced AMT with the ROCK kinase inhibitor Y-27632 or left the cells untreated. We observed 100% rounded A375m2 cells in Matrigel before addition of Y-27632. After the Y-27632 the number of rounded cells was decreased to 43%. 31% were elongated and 26% exhibited intermediate morphology (see also Additional file 1: Figure S1). Cell lysates from both untreated and treated cells were analyzed using protein microarray analysis (Kinex). 30 proteins showed significant changes in expression levels between cells cultivated without and with Y-27632. Moreover, 11 proteins exhibited significant changes in the phosphorylation of analyzed sites (Table 1). Among these proteins, we selected several candidates with potential connections to AMT-related signaling for further verification. These included β-catenin (increased expression after AMT), cofilin-2 (increase Ser3 phosphorylation after AMT) and PKCα (decreased Ser657 phosphorylation after AMT). For all the three selected proteins we confirmed the trends in changes of their phosphorylation (cofilin-2, PKCα) or expression (β-catenin) status after AMT (Figure 1). Interestingly, the changes were only seen when Y-27632 was administered in the 3D Matrigel and not in 2D conditions (Figure 1), suggesting that these changes are in fact specific for AMT. Since we were most interested in proteins with potential involvement in amoeboid invasiveness, in our next experiments we concentrated on PKCα which was found to be down-regulated after AMT.

**Table 1:** **Proteins with differential expression/phosphorylation after AMT in A375m2 cells (Kinexus)**

Protein	Phosphosite (human)	Refseq	Fold change
IKK alpha	Pan-specific	NP_001269	6.7
CDK7	Pan-specific	NP_001790	5.2
ASK1 (MAP3K5)	Pan-specific	NP_005914	3.2
STAT1a/b	Pan-specific	NP_009330	3.2
**Catenin beta**	Pan-specific	NP_001895	2.4
MEK6 (MAP2K6)	S207	NP_002749	2.3
Erk2	Pan-specific	NP_002736	2.3
**Cofilin 2**	S3	NP_068733	2.3
CASP4	Pan-specific	NP_001216	2.1
Hsp90	Pan-specific	NP_005339	-26.4
JNK1/2/3	Pan-specific	NP_002741	-22.6
Bid	Pan-specific	NP_001187	-15.2
CDK1 (CDC2)	Pan-specific	NP_001777	-9.6
PERP	Pan-specific	NP_071404	-8.7
Hsc70	Pan-specific	NP_006588	-6.1
JNK2	Pan-specific	NP_002744	-5.2
APG1	Pan-specific	NP_055093	-5.0
PDK1	Pan-specific	NP_002604	-4.3
HO1	Pan-specific	NP_002124	-3.8
**PLC gamma 1**	Y783	NP_877963.1	-3.6
CaMK1d	Pan-specific	NP_003647	-3.5
MEK4 (MAP2K4)	Pan-specific	NP_003001	-3.3
**PKC alpha**	S657	NP_002728	-3.2
IRAK2	Pan-specific	NP_001561	-3.2
Mnk2	Pan-specific	NP_060042	-3.1
p35	Pan-specific	NP_003876	-3.0
FAK	Y577	NP_005598	-3.0
BRCA1	S1497	NP_009225	-3.0
BRCA1	S1423	NP_009225	-2.9
4E-BP1	T45	NP_004086	-2.6
Bak	Pan-specific	NP_001179	-2.5
MEK1 (MAP2K1)	T385	NP_002746	-2.5
MEK5 (MAP2K5)	Pan-specific	NP_660143	-2.4
TrkA	Pan-specific	NP_002520	-2.4
Pax2	S394	NP_000269.2	-2.4
STAT3	Pan-specific	NP_003141	-2.3
MEK7 (MAP2K7)	Pan-specific	NP_005034	-2.3
PKC epsilon	S729	NP_005391	-2.3
PKBb (Akt2)	Pan-specific	NP_001617	-2.1
GCK	Pan-specific	NP_004570	-2.1
p38a MAPK	Pan-specific	NP_001306	-2.1

Antibody microarray (Kinex) screen of A375m2 cells in AMT. Cells in 3D culture were treated with ROCK inhibitor and analyzed after 24 hours. Signal values from duplicate samples were tested for significant differences with Student’s t-test followed by p-value correction for multiple testing (Benjamini-Hochberg). Proteins that showed at least two-fold change and FDR < 0.3 are shown here, complete data are available in supplementary information. Proteins discussed in the text are in boldface.

### Inhibition of PKCα results in AMT in amoeboid cells, PKC activation results in MAT in mesenchymal cells

The phosphorylation of PKCα at Ser657 is associated with increased stability of the kinase [20,21]. Our protein microarray analysis thus suggests that PKCα activation could possibly be involved in maintenance of the amoeboid morphology of melanoma A375m2 cells. To investigate the possible role of PKCα in regulating cell morphology, we analyzed the morphology of selected mesenchymal and amoeboid cell lines in 3D collagen in the presence and absence of a PKCα activator or inhibitor. The A375m2 human melanoma cell line, K2 rat sarcoma cell line and MDA-MB-231 human breast cancer cell line were used. Cells were seeded into 3D collagen matrix and the morphology of cells was observed after 48 hours of incubation. As a PKCα activator, phorbol 12-myristate 13-acetate (PMA) was used at a final concentration of 162nM. PMA is not a PKCα specific activator, but is able to activate all PKC isoforms. As a PKCα inhibitor, Gö6976 was used at a 1 μM concentration. Gö6976 is a PKCα/PKCβ specific inhibitor. However, proteomic analysis did not show any changes in other PKC isoforms than PKCα, so the activator and particularly the inhibitor were considered to be sufficiently specific for our experiments.

**Figure 2:**
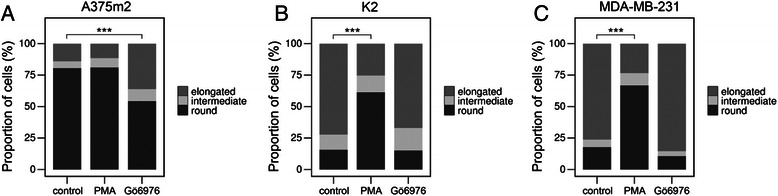
Inhibition of PKCα results in AMT in amoeboid cells, PKC activation results in MAT in mesenchymal cells. Cell morphology analysis of A375m2 **(A)**, K2 **(B)** and MDA-MB-231 **(C)** strains upon PKC activator PMA or PKCα/β inhibitor Gö6976 treatment. Cells were incubated with PMA (162nM) or Gö6976 (1 μM) for 48 hours in 3D collagen and then analyzed using NIS Elements software (Nikon). Cells were classified according to the elongation index as rounded, intermediate or elongated (see Methods section). Statistical significance (marked by asterisks) was evaluated using Pearson’s Chi-squared test. Representative results are shown out of minimum three independent experiments.

Analysis of the cellular morphology in 3D collagen confirmed that A375m2 cells are primarily amoeboid (rounded cell morphology) and K2 and MDA-MB-231 cells are primarily mesenchymal (elongated cell morphology) (Figure 2, Additional file 2: Figure S2). Upon PMA treatment, the morphology of K2 and MDA-MB-231 cells shifted from mesenchymal towards amoeboid. For K2 cells, the proportion of rounded cells was increased approximately 4 fold, and for MDA-MB-231 cells a 3 fold increase was observed (Figure 2, Additional file 2: Figure S2). In both cases, the number of elongated cells decreased proportionally. This strongly suggests the occurrence of the MAT. PMA did not have a significant effect on A375m2 cells as these cells were already of predominantly rounded morphology. In contrast, treatment with the PKCα inhibitor Gö6976 resulted in the AMT of A375m2 cells, manifested by a 2.5 fold increase in the proportion of elongated cells. Simultaneously, the proportion of rounded cells decreased. Gö6976 did not greatly affect the morphology of K2 and MDA-MB-231 cells as these cells were already of mainly elongated morphology (Figure 2, Additional file 3: Figure S3).

### Inhibition of PKCα results in decreased cancer cell invasiveness

**Figure 3:**
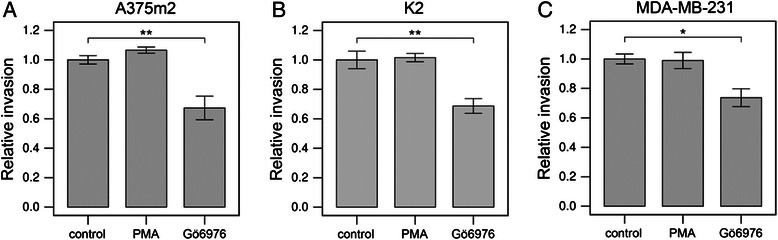
Inhibition of PKCα results in decreased invasiveness of cancer cells. Invasion analysis of A375m2 **(A)**, K2 **(B)** and MDA-MB-231 **(C)** strains upon PKC activator PMA or PKCα/β inhibitor Gö6976 treatment. Cells were plated onto 3D collagen and let to invade it for 48 hours in the presence of PMA (162nM) or Gö6976 (1 μM). 3D collagen cross-sections were analyzed using NIS Elements software (Nikon) and average invasion depth was calculated and normalized to that of untreated cells. The invasion depth in each individual field of view was assessed as sum of invasion depths of individual cells divided by the total number of cells in the respective field of view (including cells on the top of collagen). Statistical significance (marked by asterisks) was evaluated using ANOVA followed by Tukey’s honest significant difference test.

Next, the invasivity of cells in vitro was analyzed to determine the effect of PKCα on the capacity of cells to invade 3D collagen. Incubation of cells with the PKC activator PMA did not significantly change the cell invasion potential of any of the three cell lines examined. In contrast, treatment with the PKCα/β inhibitor Gö6976 resulted in a mild but significant decrease in the invasiveness of all three cell lines (Figure 3). This decrease was 1.3 fold in the case of K2 cells, 1.5 fold in the case of MDA-MB-231 cells, and 1.4 fold in the case of A375m2 cells. To exclude the possibility that the decreased invasiveness is caused by cytotoxic effect of PKC alpha inhibition, we have analyzed the effect of Gö6976 on cell survival of MDA-MB-231 cells. We found no significant effect of the inhibitor at the concentration used in our assays (1.7% and 3.3% dead cells in control and treated cells, respectively with chi-squared test p-value 0.197).

### Overexpression or activation of PKCα promotes MAT in mesenchymal cells, dominant-negative PKCα promotes AMT in amoeboid cells

To further confirm the role of PKCα in amoeboid invasiveness and AMT/MAT we decided to validate the effects of the PKC inhibitor and activator using a genetic approach. In all three cell lines we overexpressed wt PKCα or dominant-negative PKCα (K368R) to achieve activation and inhibition of PKCα, respectively. In transfected cells, the expression of each PKCα variant was demonstrated using an immunoblot (Additional file 3: Figure S3A). Surprisingly, the expression of dominant-negative PKCα was much lower than that of wt PKCα, possibly due to negative selection. The activation status of the variants was verified with a phospho-specific antibody against Thr497 in the activation loop of PKCα (Additional file 3: Figure S3A).

**Figure 4:**
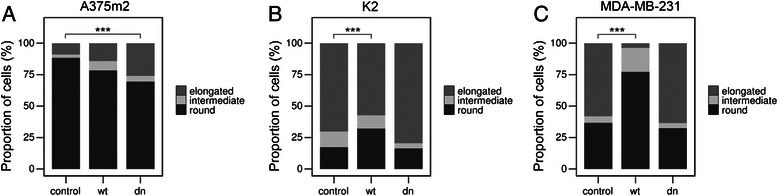
Dominant-negative PKCα promotes AMT in amoeboid cells, overexpression of PKCα promotes MAT in mesenchymal cells. Cell morphology analysis of A375m2 **(A)**, K2 **(B)** and MDA-MB-231 **(C)** strains overexpressing wt PKCα or expressing dominant-negative PKCα. Cells were transfected with pCMV6 vector expressing either wt PKCα or dominant-negative PKCα and their morphology was observed after 48 hours in 3D collagen. Cells were analyzed using NIS Elements software (Nikon). Cells were classified according to the elongation index as rounded, intermediate or elongated (see Methods section). Statistical significance (marked by asterisk) was evaluated using Pearson’s Chi-squared test. Representative results are shown out of minimum three independent experiments.

To verify the possible role of PKCα in regulating AMT/MAT, we performed morphology assays in 3D collagen with the A375m2 human melanoma cell line, K2 rat sarcoma cell line and MDA-MB-231 human breast cancer cell line transfected with PKCα variants. Upon the overexpression of wt PKCα, K2 and MDA-MB-231 cells changed their morphology towards amoeboid. For both cell lines, the proportion of rounded cells was increased approximately 1.7 fold (Figure 4, Additional file 4: Figure S4) and the number of elongated cells decreased proportionally. This strongly suggests the occurrence of MAT. The expression of wt PKCα did not have significant effect on A375m2 cells as these cells were already of predominantly rounded morphology. In contrast, the expression of dominant-negative PKCα resulted in the AMT of A375m2 cells, manifested by a 2 fold increase in the proportion of elongated cells. Simultaneously, the proportion of rounded cells decreased. The expression of dominant-negative PKCα did not greatly affect the morphology of K2 and MDA-MB-231 cells as these cells were already of mainly elongated morphology (Figure 4, Additional file 4: Figure S4).

### Expression of dominant-negative PKCα results in decreased cancer cell invasiveness

**Figure 5:**
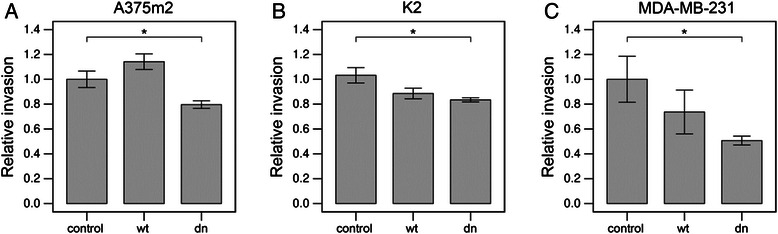
Expression of dominant-negative PKCα results in decreased invasiveness of cancer cells. Invasion analysis of A375m2 **(A)**, K2 **(B)** and MDA-MB-231 **(C)** strains overexpressing wt PKCα or expressing dominant-negative PKCα. Cells were transfected with pCMV6 vector expressing either wt PKCα or dominant-negative PKCα and let to invade into 3D collagen for 48 hours. 3D collagen cross-sections were analyzed using NIS Elements software (Nikon) and average invasion depth was calculated and normalized to that of untreated cells. The invasion depth in each individual field of view was assessed as sum of invasion depths of individual cells divided by the total number of cells in the respective field of view (including cells on the top of collagen). Statistical significance (marked by asterisks) was evaluated using ANOVA followed by Tukey’s honest significant difference test.

Next, the invasivity of cells in vitro was analyzed to determine the effect of PKCα variants on the capacity of cells to invade 3D collagen. Again, K2, A375m2 and MDA-MB-231 cell lines transfected with wt or dominant-negative PKCα were used. The expression of wt PKCα resulted in a slight, but not significant decrease of invasiveness in mesenchymal cell lines and also a non-significant increase of invasiveness in amoeboid A375m2 cells. More importantly, the expression of dominant-negative PKCα resulted in a significant decrease of invasiveness in all investigated cancer cell lines (Figure 5). These results suggest that PKCα is important for effective cancer cell invasiveness irrespective of the type of cancer cell invasion.

### siRNA-mediated downregulation of PKCα results in AMT in A375m2 amoeboid cells

**Figure 6:**
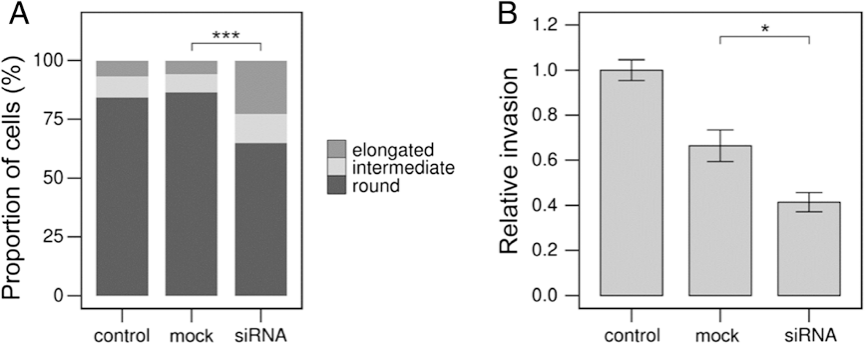
siRNA-mediated downregulation of PKCα results in AMT in A375m2 amoeboid cells. **A)** Cell morphology analysis of A375m2 strain transfected with siRNA against PKCα. Cells were transfected with siRNA and their morphology was observed after 48 hours in 3D collagen. Cells were analyzed using NIS Elements software (Nikon). Cells were classified according to the elongation index as rounded, intermediate or elongated (see Methods section). Statistical significance (marked by asterisk) was evaluated using Pearson’s Chi-squared test. Representative result is shown out of three independent experiments. **B)** Cell invasion analysis of A375m2 strain transfected with siRNA against PKCα. Cells were transfected with siRNA and let to invade into 3D collagen for 48 hours. 3D collagen cross-sections were analyzed using NIS Elements software (Nikon) and average invasion depth was calculated and normalized to that of untreated cells The invasion depth in each individual field of view was assessed as sum of invasion depths of individual cells divided by the total number of cells in the respective field of view (including cells on the top of collagen). Statistical significance (marked by asterisks) was evaluated using ANOVA followed by Tukey’s honest significant difference test.

To further confirm the specific effect of PKCα isoform on the cell morphology and invasiveness of amoeboid cancer cells, specific downregulation of PKCα expression in A375m2 cells was performed using siRNA against PKCα. In comparison to control and mock-transfected cells, total PKCα expression level in siRNA treated cells was significantly lower (Additional file 3: Figure S3B). In agreement with the effect of the PKCα inhibitor and expression of dominant-negative PKCα, siRNA treated cells exhibited a more mesenchymal morphology profile than control and mock-transfected cells, suggesting the induction of AMT. The proportion of elongated cells increased 2 fold, and there was a minor increase in the proportion of intermediate cells as well. Consistently, the proportion of rounded cells decreased (Figure 6A, Additional file 5: Figure S5). The amoeboid to mesenchymal transition was also manifested by elevated ability of siRNA treated A375m2 cells to degrade gelatin (Additional file 6: Figure S6).

In vitro invasion analysis of siRNA transfected cells showed that PKCα downregulation reduced the cell invasivity of A375m2 cells in 3D collagen (Figure 6B). Compared to mock-transfected cells, the ability of siRNA treated cells to invade into 3D collagen was reduced 1.5 fold, and this reduction was even higher (2.5 fold) compared to untreated cells. This is consistent with previous experiments where PKCα inhibition also resulted in a significant decrease in cell invasion potential. Together, these results further confirm the important role of PKCα in amoeboid invasiveness.

## Discussion

PKCα is a very important protein regulating many signaling pathways in cells, such as proliferation, differentiation, apoptosis and tumorigenesis (reviewed in [22]), and its deregulation is involved in many diseases (reviewed in [23]). PKCα has also been shown to regulate cell migration and cancer cell invasion [24-29]. Proteomic analysis of amoeboid A375m2 cells grown in 3D Matrigel revealed that phosphorylation of PKCα at Ser657 is reduced upon a ROCK kinase inhibitor Y-27632 mediated induction of AMT. Importantly, we found that the observed changes in the phosphorylation of PKCα at Ser657 and two additional candidate markers of AMT occur only in 3D Matrigel and not in 2D conditions. These results confirm the necessity of performing AMT analyses in the 3D environment.

In addition to decreased levels of PKCα phosphorylation at Ser657, the phosphoproteomics screen revealed a significant decrease in the phosphorylation of a PKCα upstream activator, PLCγ1 on Tyr783 (Table 1). This suggests that treatment with the ROCK inhibitor Y-27632 affects signaling leading to the activation of PKCα, rather than directly inhibiting PKCα.

Ser657 is located in the hydrophobic region of PKCα and its phosphorylation controls the accumulation of active PKCα and prevents its down-regulation [20,21]. The phosphoproteomics screen data thus suggested that by affecting the level of PKCα activation we could induce AMT or MAT in cancer cells. To confirm this, we analyzed the effect of PKCα inhibition and activation on the morphology of amoeboid A375m2 and two mesenchymal K2 and MDA-MB-231 cell lines. This morphology analysis revealed that the level of PKCα activation is indeed important for the regulation of cell morphology in the 3D environment. PKCα activation resulted in MAT in both mesenchymal cell lines tested and, correspondingly, PKCα inhibition or downregulation resulted in AMT in amoeboid A375m2 cells.

Next, in vitro invasivity assays into 3D collagen were performed to elucidate the effect of PKCα directly on cell invasiveness. Although PKCα activation did not significantly influence the invasivity of cells, PKCα inhibition resulted in a considerable decrease in the invading abilities of all three cell lines used (K2, A375m2 and MDA-MB-231). These results suggest that a proper level of PKCα activation is necessary for effective invasiveness of both amoeboid and mesenchymal cancer cell lines. This is consistent with previous results that have shown that PKCα regulates cell invasion and migration in several cancer cell lines. Inhibition of PKCα has decreased the invasion of urinary bladder carcinoma [30], colon carcinoma [31], renal cell carcinoma [32], breast carcinoma [33], multiple myeloma [34], glioma [35], breast cancer [36] and endometrial cancer cells [37]. PKCα activation and overexpression have been shown to increase cancer cell migration [31,33,34,36]. Consistently, PKCα knockdown leads to a decrease in cancer cell invasion [31,36,37]. Remarkably, PKCα was shown to be a marker of aggressiveness in breast cancer, as patients with PKCα-positive tumors exhibited poorer prognosis than patients with PKCα-negative tumors [36].

Since PKC alpha was shown to be involved in cancer cell survival (e.g. [38]), we had to rule out the possibility the observed decrease of invasiveness could be at least in part caused by a weak cytotoxic effect of PKCα inhibition. We found that under our conditions, PKCα inhibition does not have detectable cytotoxic effect.

Interestingly, the PKCα overexpression resulted in possibly decreased invasion (but not significant) in mesenchymal cell lines and increased invasion (also not significant) in amoeboid cell line, suggesting that for mesenchymal invasiveness endogenous level of PKCα activity could be optimal and further increase of PKCα expression may not result in increased invasion capability. Also it has to be taken into account that amoeboid migration is faster particularly under non-restrictive conditions [39]. In dense 3D collagen gels the size of the pores is limiting for non-proteolytic migration and under such conditions, tunnels formed by the mesenchymal cells could be a more effective strategy, especially in cells which are originally mesenchymal (K2) or intermediate (MDA-MB-231).

Together, these results suggest a possible role of PKCα in the amoeboid invasion of cancer cells. The setup of our proteomic screen and the effects of PKCα activity on amoeboid cancer cells suggest that PKCα might activate the small GTPase RhoA or ROCK kinase. The Rho/ROCK signaling pathway controls phosphorylation of the myosin light chain and mediates the enhanced acto-myosin contractility that is a critical feature of amoeboid cells [10,11,14]. However, it is likely that PKCα does not phosphorylate RhoA or ROCK directly; rather, PKCα might phosphorylate their upstream regulators.

Indeed, there is evidence in the literature of PKCα regulating the Rho/ROCK signaling pathway through upstream regulators, together with their relevance to cell adhesion and cytoskeletal reorganization. Earlier results showed that PKCα can be activated upon thrombin treatment and can enhance RhoA activity in endothelial cells, therefore mediating cell contraction [40,41]. PKCα phosphorylates p115RhoGEF, a specific Rho guanine nucleotide exchange factor, and increases its activity [42]. Consequently, RhoA activity is also increased and this is connected to the regulation of stress fiber formation and cell contraction [42]. One of the PKCα substrates is also RhoGDIα [43]. RhoGDIα is a cytosolic protein that binds the GDP-bound small RhoGTPases RhoA, Rac1 and Cdc42, and prevents them from the exchange of GDP to GTP [44]. PKCα phosphorylates RhoGDIα at Ser34 to reduce its affinity for RhoA (but not for Rac1 or Cdc42) [43]. So, RhoA can be activated by GTP binding and downstream signaling is enhanced, as was observed by a loss of focal adhesions [40]. Also, phosphatidylinositol bisphosphate is needed for RhoGDIα phosphorylation by PKCα; it is probably required for proper PKCα localization and orientation in the membrane [43]. Moreover, αvβ3 integrin and syndecan4-mediated PKCα activation enhances RhoA activity to form focal adhesions and maintain stress fibers [45,46].

More recently, results that connect PKCα directly to the regulation of Rho/ROCK signaling pathway were published [47-51]. PKCα regulates the localization of the small GTPase Rnd3 (also called RhoE) [47]. Rnd3 binds to ROCK kinase and prevents its downstream signaling - upon Rnd3 binding, phosphorylation of MLCP is inhibited and formation of ROCK-induced stress fibers is disturbed [48]. Moreover, Rnd3 competes with PDK1 for binding to ROCK kinase and thus prevents its proper localization to the plasma membrane [49]. PKCα phosphorylates Rnd3 and causes its translocation to internal membranes [47]. Upon this altered localization, Rnd3 is no longer able to inhibit the Rho/ROCK pathway, which is subsequently enhanced [47]. Moreover, Raf kinase pathway activation, which could be regulated by PKCα [50], was shown to be associated with Rnd3 expression [51].

Interestingly, in this study we have seen increased phosphorylation of cofilin on Ser-2 after ROCK inhibition. The positive effect of Y-27632 on cofilin phosphorylation was observed only in 3D suggesting that specific, and for AMT indispensable, condition of cultivation in 3D is responsible for the effect. Cofilin phosphorylation is regulated both by kinases (LIMK family) and phosphatases (SSH family). The KINEX microarray, however, allows to analyze only changes in expression and phosphorylation levels of LIMK1. It is hard to assess which of the pathways of cofilin phosphorylation is predominant in particular cells. We can speculate that in our case cofilin phosphorylation could be promoted via Rac/PAK1/LIMK2 signaling. In fact we see a mild (60%) increase in PAK1 expression. This was not included in our results as the p Value (0,079) did not meet our criteria for significance. Finally, though it might be contra intuitive others have also shown that Y27632 treatment can induce an increase in cofilin phosphorylation [52].

## Conclusions

Our results show that PKCα is an important regulator of the amoeboid morphology of cancer cells and its downregulation causes AMT. Furthermore, our data also indicate that PKCα is involved in both mesenchymal and amoeboid invasiveness, making it an attractive target for anti-metastatic therapies.

## Additional files

Additional file 1: Figure S1. The effect of Y-27632 treatment on morphology of A375 m2 melanoma cells. Cells were grown in 3D collagen and after 24 h cell morphology was analyzed and documented using photomicroscopy. Left panel: A375 m2 cells; right panel: A375 m2 cells treated with 10 μM Y-27632.
Additional file 2: Figure S2. The expression of PKC alpha variants and the effectiveness of PKCα silencing. A) The expression of wt PKCα, constitutively-active PKCα and dominant-negative PKCα in K2, MDA-MB-231 and A375m2 strains. Total PKCα and phosphorylation of PKCα at Thr497 were detected using corresponding antibodies in cell lysates from each strain expressing wt PKCα, constitutively-active PKCα and dominant-negative PKCα from pCMV6 vector. Representative immunoblots of each variant are shown. Actin was used as a loading control. B) The expression of total PKCα in siRNA-transfected A375m2 cells. Total PKCα level was detected in lysates from control cells (not transfected), mock cells (transfected only with transfection reagents, without siRNA) and siRNA transfected cells (siRNA against PKCα). Representative immunoblots are shown. Actin was used as a loading control.
Additional file 3: Figure S3. The effect of PKCα activation with PMA and PKCα inhibition with Gö6976 on morphology of cancer cells. The effect of PKCα activation with PMA and PKCα inhibition with Gö6976 on morphology of cancer cells. Cells were grown in 3D collagen and after 24 h cell morphology was analyzed and documented using photomicroscopy. Top panels: K2 cells; middle panels: A375 m2 cells; bottom panels: MDA-MB-231 cells. Left panels: control untreated cells; medium panels: cells treated with 162 nM PMA; right panels: cells treated with 1 μM Gö6976.
Additional file 4: Figure S4. The effect of PKCα variants expression on morphology of cancer cells. Cells were grown in 3D collagen and after 24 h cell morphology was analyzed and documented using photomicroscopy. Top panels: K2 cells; middle panels: A375 m2 cells; bottom panels: MDA-MB-231 cells. Left panels: control cells; medium panels: cells overexpressing wt PKCα; right panels: cells expressing dominant-negative version of PKCα.
Additional file 5: Figure S5. The effect of PKCα silencing on morphology of A375 m2 melanoma cells. Cells were grown in 3D collagen and after 24 h cell morphology was analyzed and documented using photomicroscopy. Left panel: A375m2 cells; medium panel: A375m2 cells treated with scrambled siRNA; right panel: A375m2 cells treated with PKCα-specific siRNA.
Additional file 6: Figure S6. The effect of PKCα on gelatin degradation by A375m2 cells. The effect of PKCα on matrix-degrading activity in A375M2 melanoma cells. **(A)** Cells were plated on fluorescent gelatin-coated coverslips and subjected to gelatin degradation assay as described in section “Methods”. Representative fields were documented by photomicroscopy. Left panel: A375M2 cells; middle panel: A375M2 cells treated with scrambled siRNA; right panel: A375M2 cells treated with PKCα -specific siRNA. Top panel: F-actin staining; bottom panel: gelatin degradation. **(B)** The quantification of matrix degrading activity was performed as described in section “Methods”. Histogram bars represent mean relative degradation obtained from 3 independent experiments and normalized to that of untreated A375M2 cells, the error bars represents standard deviations. Statistical significance was evaluated according to unpaired two-tailed Student’s t-test. Significant difference (p < 0.01) in comparison to mock-transfected cells is indicated by asterisks.

## Abbreviations

AMT: Amoeboid-mesenchymal transition
ECM: Extracellular matrix
MAT: Mesenchymal-amoeboid transition
MLC: Myosin light chain
MLCP: Myosin light chain phosphatase
PMA: Phorbol 12-myristate 13-acetate
