# Supplementary figures and images for: PKCα promotes the mesenchymal to amoeboid transition and increases cancer cell invasiveness
Source: BMC Cancer. 2015 Apr 29;15:326. doi: 10.1186/s12885-015-1347-1 (PMC4423130; doi:10.1186/s12885-015-1347-1)

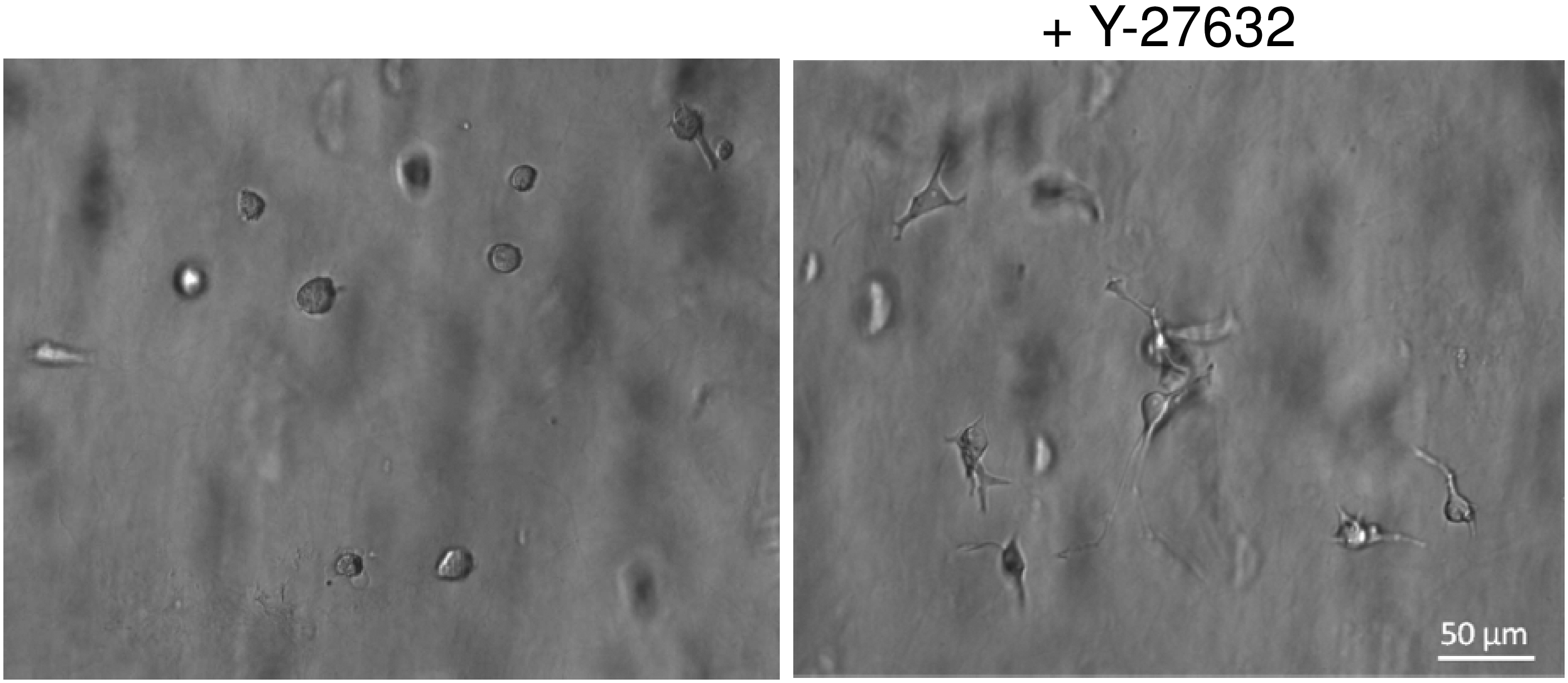

Supplement: Additional file 1: Figure S1. — The effect of Y-27632 treatment on morphology of A375 m2 melanoma cells. Cells were grown in 3D collagen and after 24 h cell morphology was analyzed and documented using photomicroscopy. Left panel: A375 m2 cells; right panel: A375 m2 cells treated with 10 μM Y-27632. [file 12885_2015_1347_MOESM1_ESM.tiff]

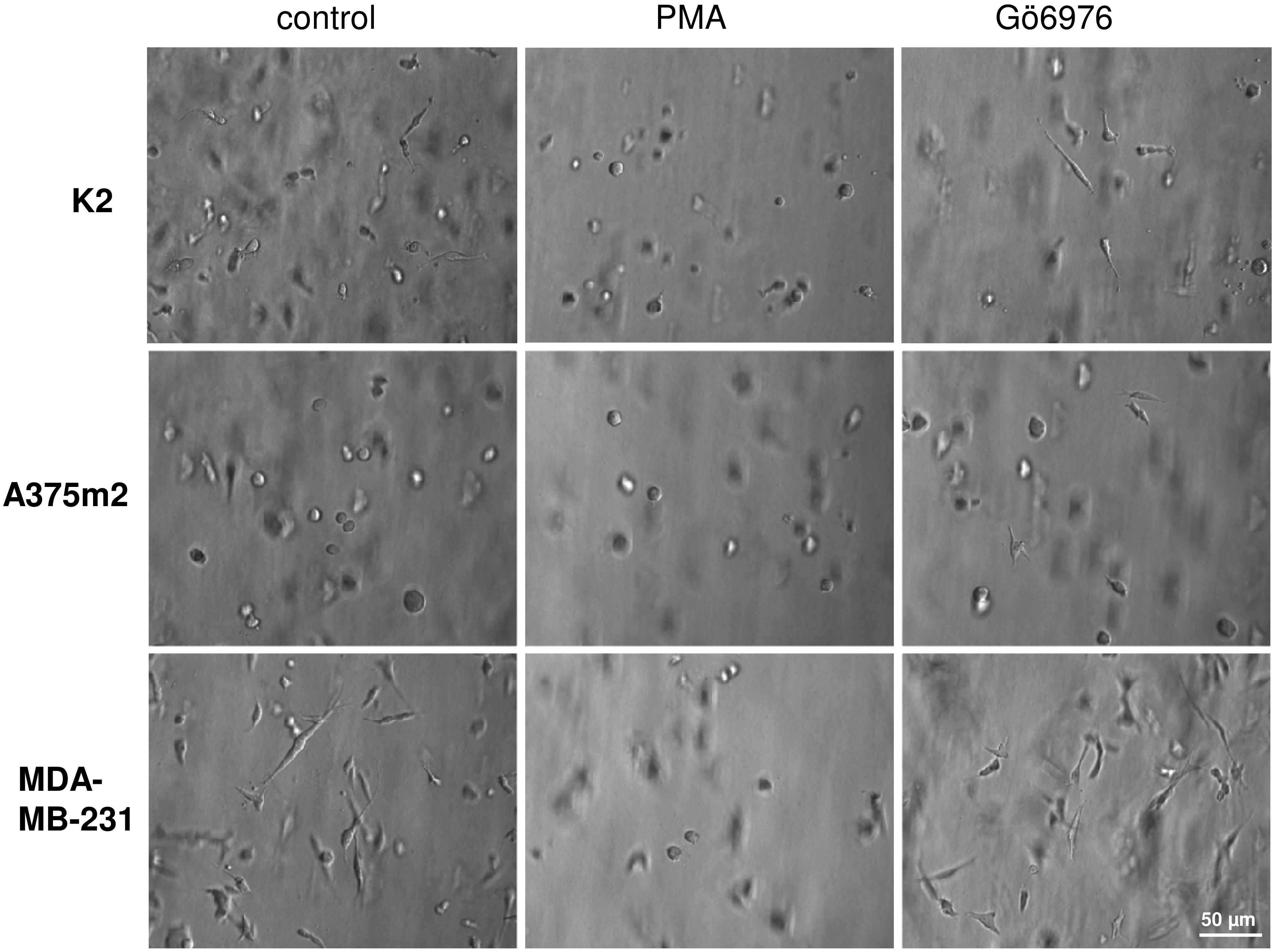

Supplement: Additional file 2: Figure S2. — The expression of PKC alpha variants and the effectiveness of PKCα silencing. A) The expression of wt PKCα, constitutively-active PKCα and dominant-negative PKCα in K2, MDA-MB-231 and A375m2 strains. Total PKCα and phosphorylation of PKCα at Thr497 were detected using corresponding antibodies in cell lysates from each strain expressing wt PKCα, constitutively-active PKCα and dominant-negative PKCα from pCMV6 vector. Representative immunoblots of each variant are shown. Actin was used as a loading control. B) The expression of total PKCα in siRNA-transfected A375m2 cells. Total PKCα level was detected in lysates from control cells (not transfected), mock cells (transfected only with transfection reagents, without siRNA) and siRNA transfected cells (siRNA against PKCα). Representative immunoblots are shown. Actin was used as a loading control. [file 12885_2015_1347_MOESM2_ESM.tiff]

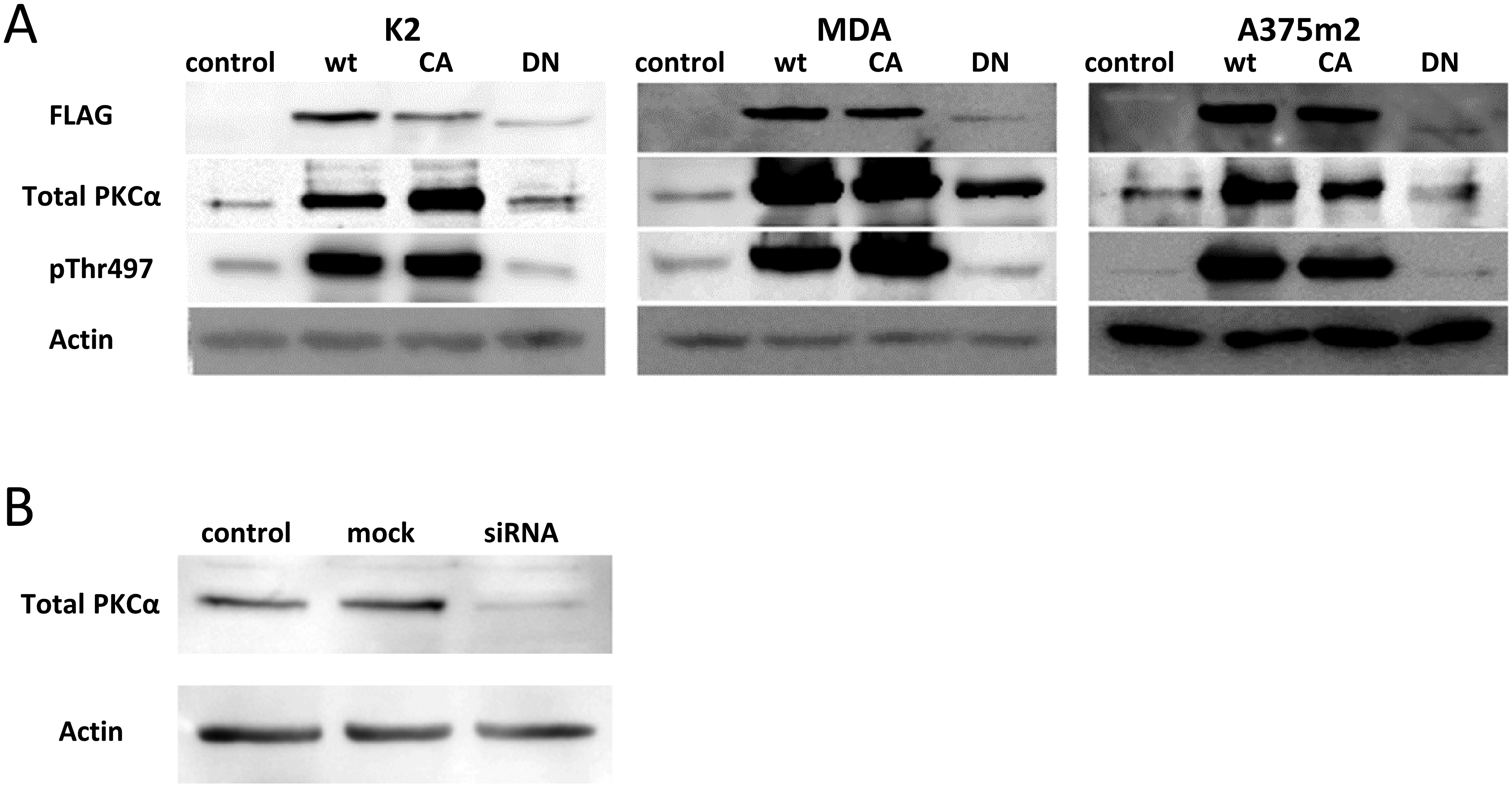

Supplement: Additional file 3: Figure S3. — The effect of PKCα activation with PMA and PKCα inhibition with Gö6976 on morphology of cancer cells. The effect of PKCα activation with PMA and PKCα inhibition with Gö6976 on morphology of cancer cells. Cells were grown in 3D collagen and after 24 h cell morphology was analyzed and documented using photomicroscopy. Top panels: K2 cells; middle panels: A375 m2 cells; bottom panels: MDA-MB-231 cells. Left panels: control untreated cells; medium panels: cells treated with 162 nM PMA; right panels: cells treated with 1 μM Gö6976. [file 12885_2015_1347_MOESM3_ESM.tiff]

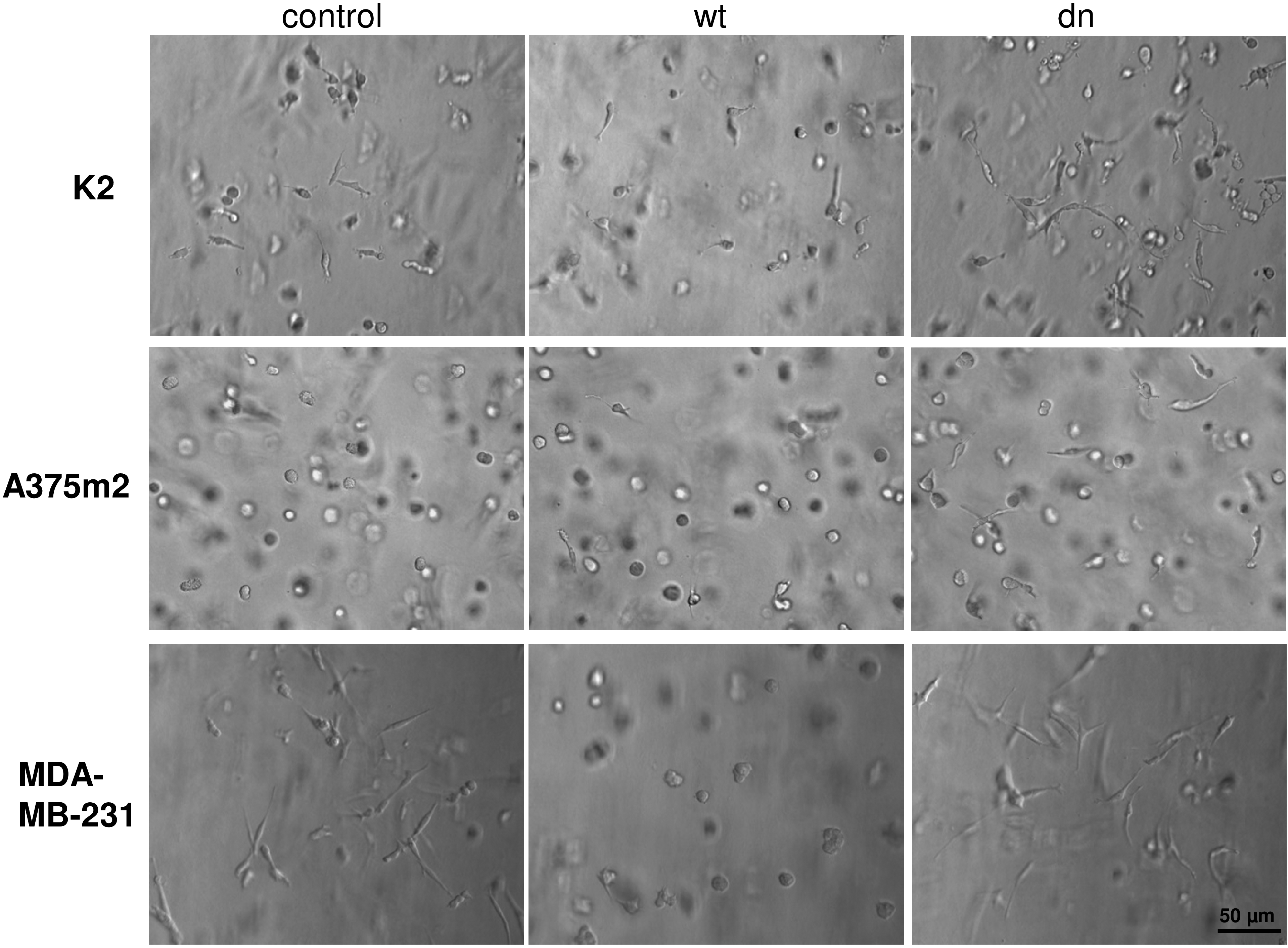

Supplement: Additional file 4: Figure S4. — The effect of PKCα variants expression on morphology of cancer cells. Cells were grown in 3D collagen and after 24 h cell morphology was analyzed and documented using photomicroscopy. Top panels: K2 cells; middle panels: A375 m2 cells; bottom panels: MDA-MB-231 cells. Left panels: control cells; medium panels: cells overexpressing wt PKCα; right panels: cells expressing dominant-negative version of PKCα. [file 12885_2015_1347_MOESM4_ESM.tiff]

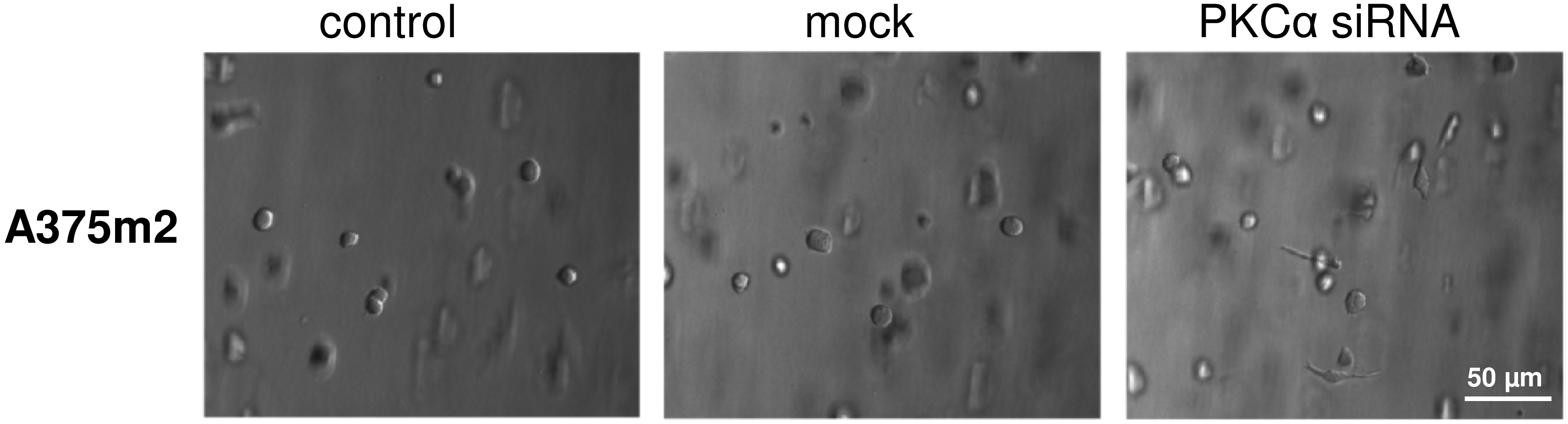

Supplement: Additional file 5: Figure S5. — The effect of PKCα silencing on morphology of A375 m2 melanoma cells. Cells were grown in 3D collagen and after 24 h cell morphology was analyzed and documented using photomicroscopy. Left panel: A375m2 cells; medium panel: A375m2 cells treated with scrambled siRNA; right panel: A375m2 cells treated with PKCα-specific siRNA. [file 12885_2015_1347_MOESM5_ESM.tiff]

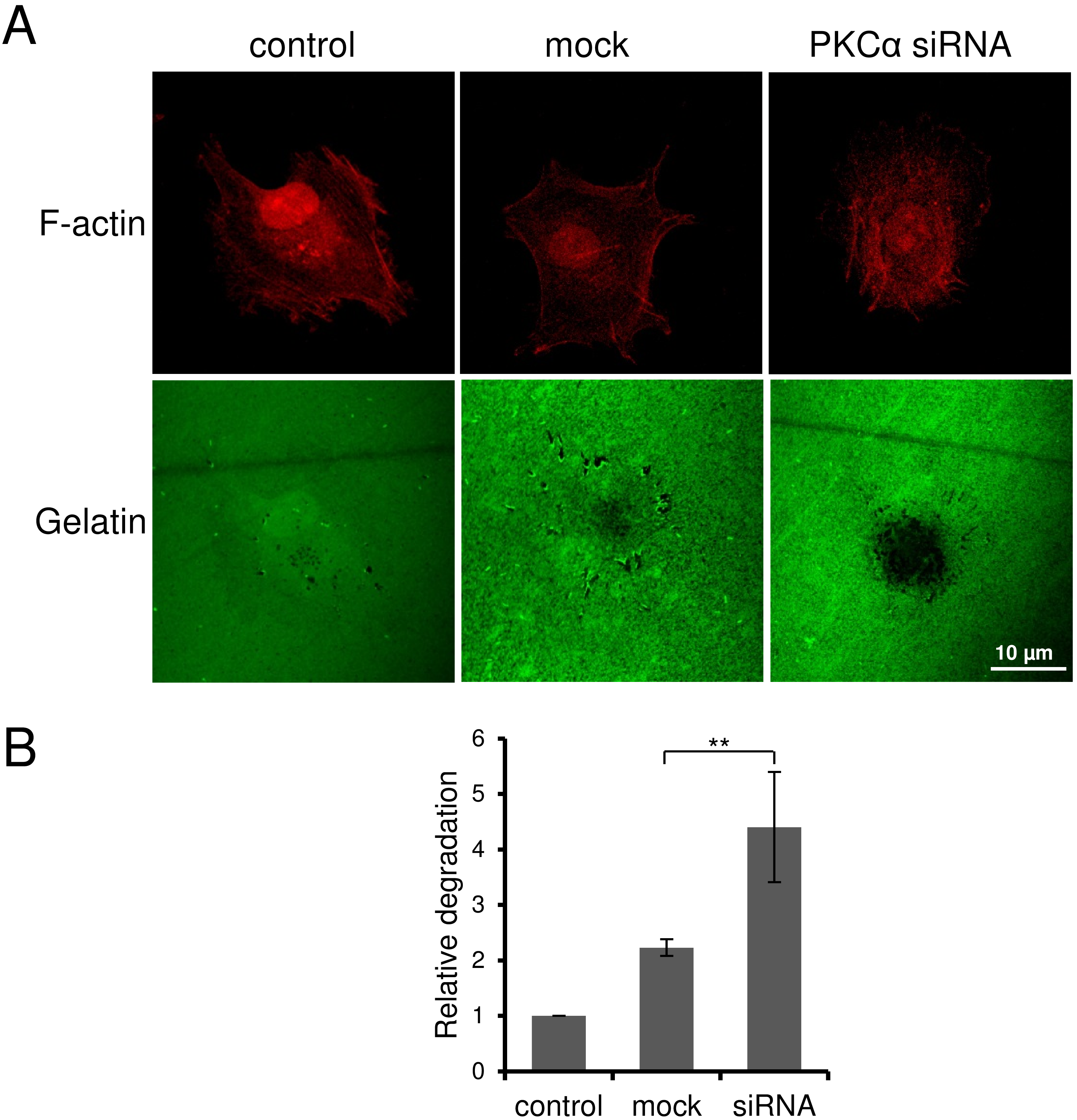

Supplement: Additional file 6: Figure S6. — The effect of PKCα on gelatin degradation by A375m2 cells. The effect of PKCα on matrix-degrading activity in A375M2 melanoma cells. (A) Cells were plated on fluorescent gelatin-coated coverslips and subjected to gelatin degradation assay as described in section “Methods”. Representative fields were documented by photomicroscopy. Left panel: A375M2 cells; middle panel: A375M2 cells treated with scrambled siRNA; right panel: A375M2 cells treated with PKCα -specific siRNA. Top panel: F-actin staining; bottom panel: gelatin degradation. (B) The quantification of matrix degrading activity was performed as described in section “Methods”. Histogram bars represent mean relative degradation obtained from 3 independent experiments and normalized to that of untreated A375M2 cells, the error bars represents standard deviations. Statistical significance was evaluated according to unpaired two-tailed Student’s t-test. Significant difference (p < 0.01) in comparison to mock-transfected cells is indicated by asterisks. [file 12885_2015_1347_MOESM6_ESM.tiff]
